# Integrating gut and IgA‐coated microbiota to identify *Blautia* as a probiotic for enhancing feed efficiency in chickens

**DOI:** 10.1002/imt2.264

**Published:** 2024-12-23

**Authors:** Chunlin Xie, Jiaheng Cheng, Peng Chen, Xia Yan, Chenglong Luo, Hao Qu, Dingming Shu, Jian Ji

**Affiliations:** ^1^ State Key Laboratory of Swine and Poultry Breeding Industry Guangdong Provincial Key Laboratory of Animal Breeding and Nutrition, Institute of Animal Science, Guangdong Academy of Agricultural Sciences Guangzhou China

## Abstract

This study explores the role of IgA‐coated bacteria in improving feed efficiency in chickens, offering a novel perspective for probiotic screening. Chickens with high feed efficiency were found to have a greater abundance of Gram‐positive bacteria, while low feed efficiency chickens exhibited higher levels of Gram‐negative bacteria and potential pathogens. Through fecal microbiota transplantation (FMT) and integrating analysis of cecal and IgA‐coated microbiota, we precisely identified *Blautia* as a key genus linked to improved feed efficiency. Further validation demonstrated that *Blautia coccoides*, a representative species of this genus, enhances feed efficiency and activates B cells to produce Immunoglobulin A (IgA), both in vivo and in vitro. Our findings provide new insights into the potential of IgA‐coated bacteria as functional probiotics, offering a promising strategy for enhancing feed efficiency in animal production.

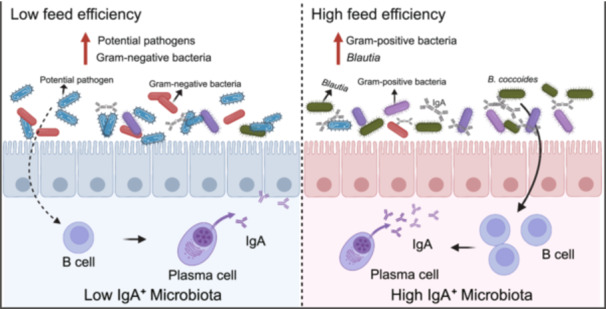

The Yellow‐feathered broiler is a local poultry breed with independent intellectual property rights in China. The Lingnan Yellow chicken series, developed by our research team, has achieved an annual distribution of over 6 million sets of parent‐generation breeding stock. This accounts for approximately 15% of the national market share. Feed efficiency is an essential trait in the livestock and poultry industries, as feed represents between 50% and 70% of total production costs [1, 2]. Therefore, improving feed efficiency is crucial for increasing profitability and ensuring the sustainability of chicken production. It has been reported that symbiotic bacteria in the intestine play a significant role in nutrient digestion and absorption, profoundly influencing feed efficiency [2, 3, 4]. However, whether restructuring microbiota can improve feed efficiency and which specific beneficial microbes are responsible for this benefit remains unclear.

Immunoglobulin A (IgA) is the most abundant antibody and plays a crucial role in microbial colonization and maintaining intestinal homeostasis [5, 6]. The secretory IgA at mucosal surfaces can directly bind to commensal bacteria, promoting their colonization and coating pathogens to facilitate their removal from the gut [7, 8, 9]. Commensal targeting by IgA shapes gut microbiota composition, regulates bacterial behaviors, modulates host physiology, and maintains metabolic homeostasis in both mice and humans [10, 11]. Recent studies have indicated that IgA‐targeted bacteria are altered in undernourished mice and children, and these IgA‐coated bacteria have a significant impact on nutrient utilization [12, 13]. Thus, investigating IgA‐coated bacteria provides a valuable perspective for understanding the role of gut microbiota in improving feed efficiency.

Over the last decade, we successfully established two distinct chicken lines with high and low feed efficiency through 15 generations of selective breeding. These lines provide unique resources for investigating the impacts of gut microbiota on feed efficiency. Here, we conducted a comprehensive comparison of the composition and development of cecal and IgA‐coated microbiota between two chicken lines. Fecal microbiota transplantation (FMT) was performed to evaluate the role of gut microbiota in feed efficiency. Additionally, integrated analyses of cecal and IgA‐coated microbiota enabled precise identification of *Blautia* as a key biomarker associated with feed efficiency. Its role in improving feed efficiency was further validated through gavage experiments in chickens and mice, complemented by in vitro studies elucidating potential underlying mechanisms. Our research provides valuable insights for identifying potential probiotics to enhance feed efficiency, particularly from the perspective of IgA‐coated bacteria.

## RESULTS AND DISCUSSION

### Comparative analysis of the composition and characteristics of gut microbiota between high‐ and low‐feed efficiency chickens

**Figure 1:**
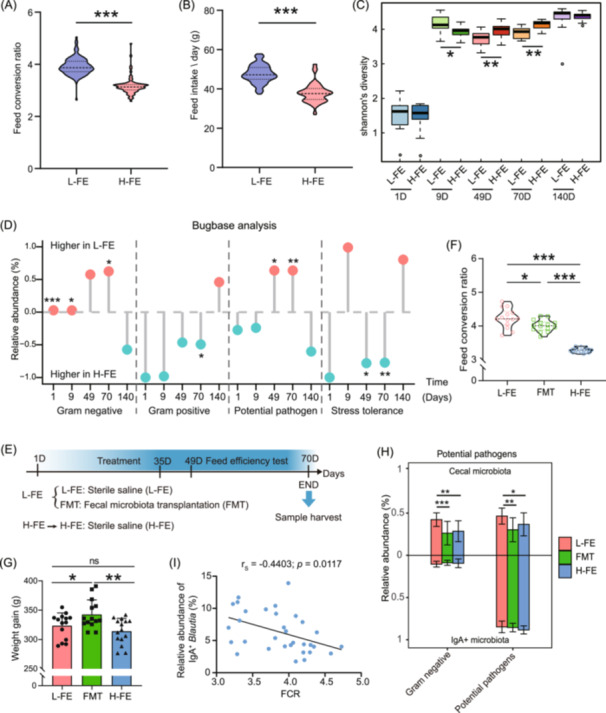
Comprehensive analysis of cecal microbiota and immunoglobulin A (IgA)‐coated microbiota between high and low feed efficiency chickens to identify biomarker bacteria associated with feed efficiency. (A, B) Feed efficiency of low (L‐FE) and high feed efficiency (H‐FE) chickens are shown as feed conversion ratio (A) and average daily feed intake (B) during Days 49 to 70 (*n* ≥ 70). Statistical significance was analyzed using an unpaired two‐tailed Student's *t*‐test. (C) Measurement of alpha diversity using Shannon's diversity index, and statistical significance was analyzed using the Wilcoxon rank‐sum test (*n* ≥ 9). Box plots are defined by the 25th and 75th percentiles; points outside this range are considered outliers (circle). (D) Microbial characteristics of L‐FE and H‐FE chickens were predicted using BugBase. Red indicates higher relative abundance in L‐FE chickens, while blue represents higher abundance in H‐FE chickens. Statistical significance was analyzed using the Wilcoxon rank‐sum test. (E) Experimental design for fecal microbiota transplantation (FMT) treatment. Low‐feed efficiency chickens received either sterile saline (L‐FE group) or microbial suspension (10^8^ colony‐forming units [CFU]/mL, FMT group), while high‐feed efficiency chickens received sterile saline (H‐FE group) via oral gavage from Day 1 to Day 35 (*n* ≥ 15). (F) Feed conversion ratio. (G) Weight gain. Statistical significance was analyzed using one‐way Analysis of Variance (ANOVA). (H) Prediction of Gram‐negative bacteria and potential pathogens in the cecal microbiome and IgA+ microbiome using BugBase. (I) Spearman correlation between the relative abundance of IgA+ *Blautia* and feed conversion ratio (FCR) value. Asterisks denote significance levels: *p < 0.05, **p < 0.01, ***p < 0.001.

The feed conversion ratio (FCR) is a widely used measure of feed efficiency. Low‐FCR animals consume less feed per unit of body weight and are considered efficient, while high‐FCR animals are deemed inefficient [14]. After 15 generations of selective breeding, low‐feed efficiency (L‐FE) chickens exhibited significantly higher FCR and average daily feed intake (ADFI) compared to high‐feed efficiency (H‐FE) chickens (*p* < 0.001, Figure1A,B). We performed 16S rRNA sequencing of cecal content at various time points. Across the five time points, L‐FE and H‐FE chickens shared 284 and 233 common operational taxonomic units (OTUs), respectively (Figure S1A,B). The shared number of OTUs between the two chicken lines increased with age (Figure S1C). Similarly, α‐diversity increased with age from Day 1 to 49. From Day 49 onward, the Chao index and observed species remained relatively stable. Interestingly, the microbiota of L‐FE chickens showed significantly higher observed species and Chao index on Day 1 (Figure S1D, E), as well as higher Shannon diversity on Day 9 (Figure 1C) compared to H‐FE chickens. In contrast, the Shannon diversity in H‐FE chickens was significantly higher than in L‐FE chickens on Days 49 and 70 (Figure 1C).

Microbial composition was shaped by age and exhibited significant differences between the two chicken lines (Figure S1F,G and Table S1). Firmicutes was the dominant phylum at Days 1 and 9, while the relative abundance of Bacteroidetes was increased and became the dominant phyla from Days 49 to 140 (Figure S1F and Tables S2, 3). Notably, on Day 1, the relative abundances of Proteobacteria, major phylum of Gram‐negative bacteria that includes a wide variety of pathogenic genera [15], were significantly higher in L‐FE chickens compared to H‐FE chickens (4.2% vs. 0.0059%, *p* = 0.0001; Table S1).

Microbial phenotype prediction using BugBase revealed that L‐FE chickens had a significantly higher relative abundance of Gram‐negative bacteria and potential pathogens. In contrast, H‐FE chickens exhibited elevated levels of Gram‐positive bacteria and enhanced stress tolerance, accompanied by reduced levels of Gram‐negative bacteria and potential pathogens (Figure 1D). Consistently, microbial functions based on Phylogenetic Investigation of Communities by Reconstruction of Unobserved States 2 (PICRUSt) revealed that the relative abundance of bacteria associated with lipopolysaccharide (LPS) biosynthesis, a component of the outer membrane in most Gram‐negative bacteria and a known trigger of inflammatory responses in the host [16], was significantly higher in L‐FE chickens compared to H‐FE chickens (Figure S2). The elevated levels of Gram‐negative bacteria and LPS biosynthesis‐associated bacteria observed in L‐FE chickens suggest a potential dysbiosis in their gut microbiota [17].

### Distinct patterns of IgA‐targeted bacteria between high‐ and low‐feed efficiency chickens

Furthermore, we measured IgA‐coated microbiota using IgA‐sequencing (IgA‐SEQ) in two chicken lines. The α‐diversity of IgA‐positive bacteria increased with age, and H‐FE chickens displayed a significantly higher number of observed species within the IgA‐coated microbiota on Day 140 (Figure S3A). Overall, the two chicken lines revealed distinct patterns of IgA‐targeted bacteria, as evidenced by significant separation in principal component analysis (PCA) (Figure S3B). Notably, the IgA‐targeted bacteria was not solely dependent on their relative abundance within the cecal microbiota. For example, Firmicutes emerged as the predominant phylum within the IgA‐coated microbiota at 49 days, whereas Bacteroidetes dominated the cecal microbiome at the same time point (Figure S3C). Additionally, the cecal microbiota of L‐FE chickens revealed a higher relative abundance of potential pathogens; however, this was not reflected in the IgA‐coated microbiota on Days 49 and 70. Conversely, on Day 140, IgA‐coated potential pathogens were significantly more abundant in H‐FE chickens compared to L‐FE chickens, despite no significant differences in the cecal microbiota (Figure S3D).

### Identification of biomarker bacteria associated with feed efficiency through integrated analysis of cecal and IgA‐coated microbiota following fecal microbiota transplantation

To investigate the role of gut bacteria in feed efficiency, we performed FMT from H‐FE chickens to L‐FE chickens (Figure 1E). As expected, FMT significantly improved feed efficiency (lower FCR) and growth performance (higher weight gain) in L‐FE chickens (Figure 1F,G), indicating that microbiota intervention is an effective strategy for enhancing feed efficiency in poultry production.

Then, we investigate whether FMT alters the composition and function of gut microbiota and IgA‐coated microbiota. Our results revealed that FMT alters the cecal microbial composition and diversity in L‐FE chickens to mirror those of H‐FE chickens (Figure S4A,B). Interestingly, the relative abundance of Gram‐positive Actinobacteria was significantly higher in H‐FE and FMT chickens than in L‐FE chickens (*p* < 0.05), while the abundance of Gram‐negative Proteobacteria decreased after FMT (Figure S4C). In the IgA‐coated microbiota, Bacteroidetes, Synergistetes, and Tenericutes were more abundant in H‐FE and FMT chickens than in L‐FE chickens (Figure S4D). BugBase predictive analysis revealed that the abundance of Gram‐negative bacteria and potential pathogens in the cecal microbiota was significantly higher in L‐FE chickens compared to H‐FE and FMT chickens. However, no significant differences were observed among the three groups in the IgA‐targeted bacteria (Figure 1H). This finding suggests that IgA in L‐FE chickens may have a lower affinity for binding pathogenic microbes.

To more precisely identify biomarker species associated with feed efficiency, we comprehensively analyzed the cecal and IgA‐coated microbiota following FMT. We hypothesized that the core functional taxa would be those microbes that are significantly more abundant in both FMT and H‐FE chickens compared to L‐FE chickens. At the genus level, the cecal microbiota in FMT and H‐FE chickens had higher abundances of *Peptococcus*, *Dorea*, and *Blautia*, and lower levels of *Coprococcus* and *Sutterella* compared to L‐FE chickens (Figure S4E). Similarly, the IgA‐coated bacteria in FMT and H‐FE chickens were enriched with *Prevotella*, *YRC22*, *Enterococcus*, and *Blautia*, while *Megamonas* and *Coprococcus* were less abundant compared to L‐FE chickens (Figure S4F). We conducted linear discriminant analysis (LDA) to identify biomarker bacteria associated with feed efficiency (threshold ≥ 2). Consequently, *Peptococcus*, *Dorea*, and *Blautia* were identified as biomarker bacteria in the cecal microbiota, while *Blaitia* was also identified as a biomarker in the IgA‐coated microbiota (Figure S5). Among these bacterial genera, only *Blautia* emerged as a co‐biomarker in both the cecal and IgA‐coated microbiota (Figure S6). Additionally, a significant negative correlation was observed between the relative abundance of IgA‐coated *Blautia* and FCR (Figure 1I). Thus, through integrated analysis of cecal and IgA‐coated microbiota, we precisely identified *Blautia* as the biomarker genus associated with feed efficiency.

Given that IgA is produced by activated B cells derived from the bursa in birds, we examined the size of lymphoid follicles and the proportion of Bu‐1^+^ B cells in the bursa of Fabricius. The data indicated that FMT significantly enhanced both parameters, bringing their levels closer to those observed in H‐FE chickens (Figure S7A,B).

### Changes in feed efficiency and IgA production in response to colonization with *B. coccoides* in mice and chickens

**Figure 2:**
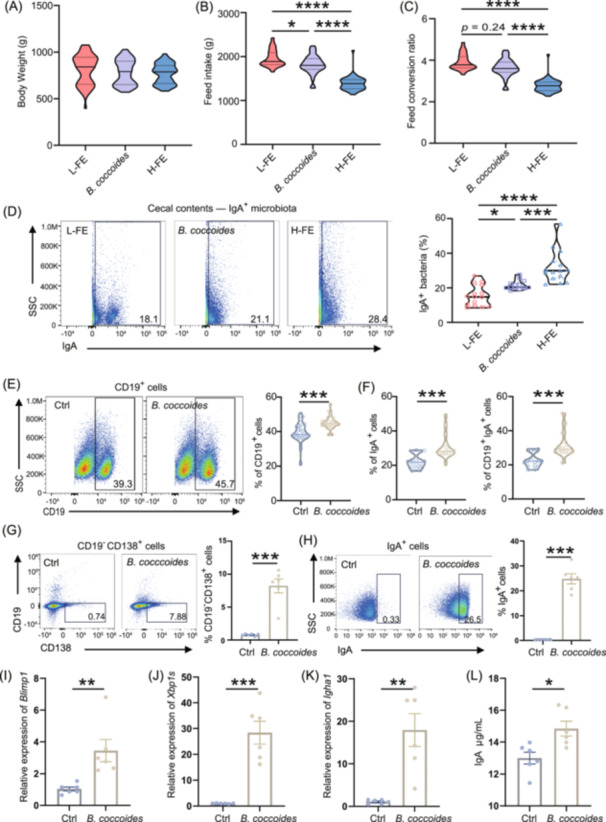
Demonstrated the role of *Blautia* as a potential probiotic in improving feed efficiency and activating B cells for IgA production in vivo and in vitro. (A) Body weight of chickens at day 70. (B) Feed intake, from Days 49 to 70. (C) Feed conversion ratio, from Days 49 to 70. (D) The proportion of IgA^+^ bacteria in cecal contents analyzed by flow cytometry (*n* = 15). (E, F) Frequency of CD19^+^ B cells, IgA^+^ cells, and CD19^+^ IgA^+^ cells of mesenteric lymph nodes analyzed by flow cytometry (*n* = 15). (G, H) Flow cytometry analysis illustrates the frequency of CD19^−^ CD138^+^ plasma cells and IgA^+^ cells following 3 h coculture of the B‐cell line Ramos with *B. coccoides* in vitro (*n* = 6). (I–K) Gene expression of B lymphocyte‐induced maturation protein‐1 (*Blimp1*), X‐box binding protein 1 (*Xbp1s*), and immunoglobulin heavy constant alpha 1 (*Igha1*) (*n* = 6). (L) The IgA concentration was analyzed using enzyme‐linked immunosorbent assay (ELISA). Statistical significance between two groups were performed using an unpaired, two‐tailed Student's *t*‐test, while one‐way ANOVA was applied for comparisons among three groups. Asterisks denote significance levels: *p < 0.05, **p < 0.01, ***p < 0.001, ****p < 0.0001. SSC, side scatter.

*Blautia* is considered as a new functional genus with potential probiotic properties, such as improving metabolic disorders by metabolizing tryptophan into indole‐3‐acetic acid and preventing inflammation by upregulating intestinal regulatory T cells [18, 19]. However, its impact on feed efficiency remains unclear. Therefore, we administered *Blautia coccoides*, a potential probiotic with a significant role in regulating host energy metabolism and mucus immunity of the genus *Blautia* [19, 20], to newborn low feed efficiency chicks and weaned C57BL/6j mice respectively (Figure S7C,D). We found that *B. coccoides* did not affect the body weight of chickens and mice (Figures 2A, S7E), but significantly reduced the feed intake of L‐FE chickens (Figure 2B). Moreover, *B. coccoides* showed potential in improving feed efficiency in L‐FE chickens and mice (Figures 2C, S7F). The FCR of L‐FE chickens decreased from 3.86 to 3.75 following *B. coccoides* administration. A notable increase in IgA‐coated bacteria was observed in the cecal contents after *B. coccoides* administration in both chickens and mice (Figures 2D, S7G). Additionally, significantly elevated levels of CD19^+^ cells, IgA^+^ cells, and CD19^+^ IgA^+^ cells were observed in the mesenteric lymph nodes of mice following *B. coccoides* treatment (Figure 2E,F). These results suggested that *B. coccoides* has the potential to improve feed efficiency and increase the activation of B cells.

To further investigate the underlying mechanisms, we cocultured the B‐cell line Ramos with *B. coccoides* in vitro, revealing that *B. coccoides* significantly stimulated B cell activation, leading to a noticeable increase in the IgA‐producing CD19^‐^ CD138^+^ plasma cells and IgA^+^ cells (Figure 2G,H). This was accompanied by upregulation of genes associated with B cell activation (B lymphocyte‐induced maturation protein‐1, *Blimp1* and X‐box binding protein 1, *Xbp1s*) and IgA production (Immunoglobulin heavy constant alpha 1, *Igha1*) (Figure 2I–K), along with a notable elevation in IgA secretion (Figure 2L). Similarly, primary B cells isolated from 49‐day‐old chickens showed enhanced viability and activation when cocultured with *B. coccoides*, as evidenced by an increased proportion of IgA^+^ B cells and elevated IgA secretion (Figure S8). While previous studies have highlighted the role of *Blautia* in T lymphocyte development, its impact on B cells and IgA production had not been reported until now. Further studies utilizing IgA‐knockout animals will be crucial to elucidate the underlying mechanisms of the IgA‐*Blautia* interactions in improving feed efficiency.

## CONCLUSION

In summary, we demonstrated that H‐FE chickens had a greater abundance of Gram‐positive and stress‐tolerant strains, while L‐FE chickens exhibited an increased abundance of harmful bacteria, including Gram‐negative microbes and potential pathogens. By integrating analysis of cecal and IgA‐coated microbiota, we precisely identified *Blautia* as a biomarker bacterium, potentially enhancing feed efficiency through the activation of B cells to produce IgA. Thus, our study offers a precision approach to identifying functional probiotics and provides a new strategy for improving feed efficiency in animal production.

## METHODS

Sample numbers for 16S rRNA sequencing are listed in Table S4. Detailed procedures for FMT, *B. coccoides* administration in chickens and mice, coculture of B cells with *B. coccoides*, sample collection, 16S rRNA sequencing, bioinformatics, and statistical analysis methods are provided in the Supplementary Information.

## AUTHOR CONTRIBUTIONS

**Chunlin Xie**: Writing—original draft; funding acquisition; investigation; visualization; formal analysis; validation; writing—review and editing. **Jiaheng Cheng**: Investigation; validation. **Peng Chen**: Investigation; validation. **Xia Yan**: Investigation; validation. **Chenglong Luo**: Conceptualization; writing—review and editing. **Hao Qu**: Conceptualization; writing—review and editing. **Dingming Shu**: Conceptualization; funding acquisition; writing—review and editing. **Jian Ji**: Conceptualization; funding acquisition; writing—review and editing.

## CONFLICT OF INTEREST STATEMENT

The authors declare no conflicts of interest.

## ETHICS STATEMENT

All experimental procedures were performed in accordance with the guidelines for the Institutional Animal Care and Use Committee of the Institute of Animal Science, Guangdong Academy of Agricultural Sciences (Approval Nos. 2023007 and Q021), Guangzhou, China.

## Supporting information


**Figure S1.** Dynamic alterations in the microbial community during the growth of L‐FE and H‐FE chickens.
**Figure S2.** Differential functions analysis of the cecal microbiota based on PICRUSt prediction between L‐FE and H‐FE chickens.
**Figure S3.** Composition and function of IgA‐coated microbiota between L‐FE and H‐FE chickens.
**Figure S4.** Effects of FMT on cecal and IgA‐coated microbial diversity and composition.
**Figure S5.** Identification biomarker bacteria associated with feed efficiency.
**Figure S6.** Boxplots display the relative abundance of shared bacterial taxa identified by LDA score analysis.
**Figure S7.** FMT on chickens and *B. coccoides* administration in chickens and mice.
**Figure S8.** Primary B cells of chicken co‐culture with *B. coccoides*.

**Table S1.** The significant differences of microbial composition at the phylum and genus levels.
**Table S2.** Relative abundance of cecal microbiota at the phylum level.
**Table S3.** Relative abundance of cecal microbiota at the genus level.
**Table S4.** Sample number for 16S rRNA sequencing.

## Data Availability

The details of material and methods are available in the supplementary materials. Raw data for 16S rRNA gene sequencing for cecal and IgA‐coated microbiome can be accessed at Sequence Read Archive (SRA) database with accession number PRJNA994595 (https://www.ncbi.nlm.nih.gov/sra/?term=PRJNA994595). The data and scripts used are saved in GitHub https://github.com/cylinqueen/chicken-micro. Supplementary materials (methods, figures, tables, graphical abstract, slides, videos, Chinese translated version, and update materials) may be found in the online DOI or iMeta Science http://www.imeta.science/.
