# Supplementary material for: Integrating gut and IgA‐coated microbiota to identify Blautia as a probiotic for enhancing feed efficiency in chickens
Source: Imeta. 2024 Dec 23;4(1):e264. doi: 10.1002/imt2.264 (PMC11865324; doi:10.1002/imt2.264)
Supplement: Supplementary file 1 — Figure S1. Dynamic alterations in the microbial community during the growth of L‐FE and H‐FE chickens. Figure S2. Differential functions analysis of the cecal microbiota based on PICRUSt prediction between L‐FE and H‐FE chickens. Figure S3. Composition and function of IgA‐coated microbiota between L‐FE and H‐FE chickens. Figure S4. Effects of FMT on cecal and IgA‐coated microbial diversity and composition. Figure S5. Identification biomarker bacteria associated with feed efficiency. Figure S6. Boxplots display the relative abundance of shared bacterial taxa identified by LDA score analysis. Figure S7. FMT on chickens and B. coccoides administration in chickens and mice. Figure S8. Primary B cells of chicken co‐culture with B. coccoides. [file IMT2-4-e264-s002.docx]

**Supplementing information to**

**Integrating Gut and IgA-Coated Microbiota to Identify Blautia as a Probiotic for Enhancing Feed Efficiency in Chickens**

**Runing title:** Potential probiotic associated with feed efficiency

Chunlin Xie, Jiaheng Cheng, Peng Chen, Xia Yan, Chenglong Luo, Hao Qu, Dingming Shu*, Jian Ji*

State Key Laboratory of Swine and Poultry Breeding Industry, Guangdong Provincial Key Laboratory of Animal Breeding and Nutrition, Institute of Animal Science, Guangdong Academy of Agricultural Sciences, Guangzhou 510640, China.

*Correspondence: [jijian1017@163.com](mailto:jijian1017@163.com) (Jian Ji) and [shudm@263.net](mailto:shudm@263.net) (Dingming Shu)

Supplementary Materials and Methods

**Animals and sample collection**

The animal study and sample collection were cared for and used according to the humane requirements of the Institute of Animal Science, Guangdong Academy of Agricultural Sciences. Furthermore, all experimental procedures were performed in accordance with the guidelines for the Institutional Animal Care and Use Committee of the Institute of Animal Science, Guangdong Academy of Agricultural Sciences (Approval No. 2023007 and Q021), Guangzhou, China.

The feed conversion ratio (FCR), defined as the amount of feed consumed per unit of weight gain, is a widely used measure of feed efficiency. Low-FCR animals consume less feed per unit of body weight and are considered efficient, while high-FCR animals are deemed inefficient [1]. Huiyang Bearded chicken, one of the most famous Yellow-feathered broiler breeds known for its superior meat quality, has been utilized for long-term breeding. The selective breeding of high feed efficiency (H-FE) and low feed efficiency (L-FE) lines in Huiyang Bearded chickens was established as previously described [2]. Briefly, average selection pressure in males and females was 10 % and 50 %, respectively. In each generation, approximately the top 20 males and 100 females with the lowest and highest feed efficiencies were retained to produce the subsequent generation. All chickens had ad libitum access to feed and water and were fed a starter diet (200 g/kg CP and 2900 kcal ME/kg) from hatching to 35 days of age, followed by a grower diet (180 g/kg CP and 2950 kcal ME/kg). The chickens in the two lines were weighed weekly, and their feed intake was recorded from day 49 to 70. The FCR value was obtained by calculation. After 15 generations of selection, we obtained two chicken lines with distinct feed efficiency, which provide unique animal models for us to investigate the correlation between microbiota and feed utilization.

To investigate differences in the composition and development of cecal and immunoglobulin A (IgA)-coated microbiota between the two chicken lines, cecal contents were collected on days 1, 9, 49, 70, and 140 for 16S rRNA sequencing and IgA-sequencing (IgA-SEQ). Samples were obtained from 15 female chickens at each time point. Feed intake and body weight gain were recorded from days 49 to 70 (*n* ≥ 70), and FCR value was calculated using the following formula:

$$\text{F}\text{CR}\text{=}\frac{\text{F}\text{eed intake (g)}}{\text{B}\text{ody weight gain (g)}}$$

**Bacterial** **flow cytometry**

IgA-coated (IgA^+^) bacteria were isolated using a combination of magnetic activated cell sorting (MACS) and fluorescence activated cell sorting (FACS). Specifically, thawed cecal content was homogenized via bead beating, and the supernatants containing fecal bacteria were collected and washed with 1 mL of phosphate buffer solution (PBS) containing 1% bovine serum albumin (BSA, Sigma-Aldrich, St. Louis, MO). A portion of mixture was used for enzyme-linked immunosorbent assay (ELISA) analysis of cecal IgA content. After centrifugation at 8000 × *g* for 5 min, the supernatant was removed, and the precipitate was resuspended in PBS containing 1% BSA.

Samples were stained with SYTO-BC (Invitrogen S-34855, 1:4000 dilution) for bacterial DNA and Mouse Anti-Chicken IgA-BIOT (Southern Biotech). The mixture was incubated at 4 ℃ for 20 min, centrifuged for 5 min at 1000 × *g*, and then resuspended in 500 μL of streptavidin-APC (1:800 final concentration) in PBS with 0.25% BSA for 15 min. The suspension was washed and centrifuged for 5 min at 1000 × *g* before being resuspended in 500 μL MACS buffer and mixed with Anti-APC MicroBeads (1:50; Miltenyi) at 4 ℃ for 15 min. Following this, the mixture was washed, centrifuged for 5 min at 1000 × *g*, and resuspended in 1 mL MACS buffer. The suspension was processed on an autoMACS separator. The fraction that did not bind to the magnet was classified as IgA^-^ bacteria. Both the IgA^+^ and IgA^-^ eluates were collected and analyzed using flow cytometry.

**Microbial genomic DNA extraction and 16S rRNA gene sequencing**

Microbial genomic DNA was isolated from cecal content and IgA^+^ eluates using the QIAamp DNA Stool Mini Kit (QIAGEN, Hilden, Germany). Details of the sample numbers for 16S rRNA sequencing in each group are provided in Table S4. DNA samples were amplified using primers (forward: 5′-GTG CCA GCMGCC GCG GTAA-3′ and reverse: 5′-GGA CTA CHVGGG TWT CTAAT-3′) targeting the V4 region of the 16S rDNA gene. Amplicons were purified using GeneJET gel extraction kit (Thermo Fisher Scientific, Carlsbad, CA) and quantified with the Qubit dsDNA Assay (Thermo Fisher Scientific). Samples were subsequently loaded onto the Illumina MiSeq platform for sequencing, following the manufacturer’s instructions (Illumina, San Diego, CA). Raw sequence data were deposited in the Sequence Read Archive (SRA) database under accession code PRJNA994595 (<https://www.ncbi.nlm.nih.gov/sra/?term=PRJNA994595>).

**16S rRNA sequencing data analysis**

Paired-end reads were generated and filtered to remove the reads with sequencing adapters, N bases, poly bases, and low-quality using default parameters. Clean paired-end reads with at least 10 bp overlaps were merged into tags using the Connection Overlapped Pair-End (COPE) software (v1.2.1) [3]. Tags were clustered into Operational Taxonomic Units (OTUs) at a 97 % similarity threshold using UPARSE (v7.0.1090) [4], and unique OTU representative sequences were obtained. Chimeras were removed using UCHIME (v4.2.40) [5]. All tags were mapped to each OTU representative sequence using USEARCH GLOBAL, and the number of tags for each OTU in each sample was summarized in an OTU abundance table. OTU representative sequences were taxonomically classified using the Ribosomal Database Project (RDP) Classifier v.2.2, trained on the Greengenes database, with a 0.8 confidence value cutoff [6]. OTU abundances were used to generate Venn diagrams with the VennDiagram package in R (v3.4.1). Alpha diversity was calculated using Mothur (v1.31.2), and means and standard deviations (SD) were determined from alpha diversity values of all samples. Significant differences in alpha diversity among groups were indicated by *p*-values less than 0.05. Beta diversity analysis based on Bray-Curtis distances was performed using QIIME (v1.80) [7]. Principal component analysis (PCA) was conducted with the “FactoMineR” package, and distance matrices were visualized using R (v3.4.1). Wilcoxon rank-sum test was used to identify significantly different taxonomic bacteria between sample groups. *p*-values were adjusted with a Benjamini-Hochberg false discovery rate correction using the ‘p.adjust’ function in R (v3.4.1). The number of tags for each taxonomic rank (Phylum and Genus) in different samples were visualized in ternary diagrams or histograms using R (v3.4.1). Microbial functions were predicted using Phylogenetic Investigation of Communities by Reconstruction of Unobserved Species (PICRUSt) as previous description [8]. Gene family mapping results were used to calculate the abundance of Kyoto Encyclopedia of Genes and Genomes (KEGG) pathways. Statistical analysis and visualization of altered KEGG pathways were conducted using the Statistical Analysis of Metagenomic Profiles (STAMP) software (<https://beikolab.cs.dal.ca/software/STAMP>), applying a two-sided White’s non-parametric *t*-test. Phenotypes of cecal and IgA-coated microbiota were predicted by BugBase (<https://bugbase.cs.umn.edu/>). OTUs were summarized at different levels and input into the Linear Discriminant Analysis (LDA) program to identify biomarkers enriched in groups of interest. An LDA score threshold of ≥ 2 was applied for biomarker selection.

**Fecal microbiota transplantation**

Fecal samples for Fecal microbiota transplantation (FMT) were collected from 30 H-FE female chickens at 70 days of age. Fresh feces, excluding the white part of the excreta, were obtained in the morning and stored in liquid nitrogen. These samples were homogenized with 0.9% sterile saline at a 1:2 ratio and subsequently filtered through sterile gauze. Following this, sterile glycerol was added to the suspension at a 1:9 ratio (1 mL sterile glycerol for 9 mL suspension), and the resulting microbial suspension was preserved at −80 ℃ until further use. Before use, the stored suspension was thawed, and methylene blue staining was employed to enumerate live microbes. The suspension was then diluted to a concentration of 10^8^ colony-forming units (CFU)/mL using sterile saline.

For the FMT experiment, 30 L-FE female post-hatched chicks were randomly assigned to two groups (L-FE group and FMT group), with 15 chicks per group. Additionally, 15 post-hatch H-FE female chicks were selected as a positive control group, with each chicken housed in an individual cage. Chicks in the FMT group received with 1 mL microbial suspension (10^8^ CFU/mL), while those in the L-FE and H-FE groups were administered sterile saline via oral gavage every other day from day 1 to 35. Body weight gain and feed intake were recorded from day 49 to 70 to compute the FCR. At 70 days, all chickens were euthanized, and serum was collected for IgA analysis. Cecal content was flash-frozen in liquid nitrogen for 16S rRNA sequencing and IgA-SEQ as descript above. The bursa of Fabricius was fixed in 4% paraformaldehyde solution for hematoxylin and eosin (H&E) staining.

**Hematoxylin and eosin (H&E) staining**

Bursa samples were fixed in 4% paraformaldehyde and embedded in paraffin. Paraffin-embedded sections (5 μm) were dewaxed, rehydrated, and stained with hematoxylin and eosin for histological examination. Images were captured using light microscopy, and lymphoid follicle sizes were measured using Image-Pro Plus software (version 6.0, Rockville, MD).

**Oral gavage of *Blautia coccoides* in C57BL/6j mice and chickens**

*B. coccoides* (ATCC 29236) was obtained from the American Type Culture Collection (ATCC) and cultured in Gifu Anaerobic medium (HB8518-1, Qingdao Hope Bio, China) at 37 ℃ in an anaerobic incubator (Defendor AMW1000, Hariolab, China).

Newborn chicks (1-day-old) with high and low feed efficiency were allocated into L-FE group, *B. coccoides* group, and H-FE group, *n* ≥ 25 per group. Chicks in the *B. coccoides* group received 200 µL of *B. coccoides* suspension (10^8^ CFU/mL) every other day, while those in the L-FE and H-FE groups received sterile saline via oral gavage every other day from days 1 to 35. All chickens were allowed *ad libitum* access to food and water. Feed intake and body weight were recorded from days 35 to 70, and all chickens were slaughtered at the end of the experiment after a 12-hour fast. Cecal contents were collected for flow cytometry analysis.

Fifty SPF female C57BL/6j mice (3-week-old) were randomly allocated into Control and *B. coccoides* groups. Each group consisted of 5 replicates with 5 mice per replicate. Mice in Control group was orally inoculated with 200 µL sterile saline (Ctrl), while mice in *B. coccoides* group were orally administrated with 200 µL of *B. coccoides* suspension every other day for 3 weeks, with a concentration of 10^8^ CFU/mL. All mice were allowed *ad libitum* access to food and water. Body weight and feed intake were recorded weekly, and the FCR value was calculated. At the end of experiment, mice were sacrificed, cecal contents were collected for assaying the proportion of IgA^+^ bacteria, and mesenteric lymph nodes were sampled for flow cytometry.

**B-cell line Ramos co-culture with *B. coccoides***

The B-cell line Ramos was cultured in Roswell Park Memorial Institute (RPMI) 1640 medium supplemented with 10% FBS and 1% Penicillin-Streptomycin in an incubator set at 37 °C with an atmosphere of 95% air and 5% carbon dioxide. B-cell suspension without Penicillin-Streptomycin was seeded onto a 12-well plate at the concentration of 10^6^ cells per well. Live *B. coccoides* was added dropwise into the plates at concentration of 4 × 10^6^ per well. At 3 h post-co-culture, B cells were collected for activation detection using Flow cytometry and qPCR. The supernatants were utilized for IgA assay via ELISA.

**Primary B cell isolation and co-culture with *B. coccoides***

Primary B cells were isolated from the bursa of Fabricius of chickens at 49-day-old. Briefly, the bursa of Fabricius was excised, sliced, and digested in dissociation buffer containing Hank’s Balanced Salt Solution (HBSS, Gibco), 10% FBS, 5 mM EDTA, and 15 mM HEPES at 37 °C for 30 min. The digested tissue was then passed through a 70 μm filter to obtain a single-cell suspension. The cells were washed three times in ice-cold PBS and subsequently surface-stained with FITC-conjugated mouse anti-chicken Bu-1 antibody (8395-31, Southern Biotech) at 4 °C for 20 min.

Bu-1^+^ B cells were sorted using a FACSAria™ Fusion (BD Biosciences) cell sorter. Sorted primary B cells were plated in 12-well plates at a density of 1 × 10^6^ cells per well in RFMI 1640 medium supplemented with 10% FBS (Thermo Fisher Scientific) and penicillin-streptomycin (Thermo Fisher Scientific). Live *B. coccoides* was added to each well at a concentration of 4 × 10^6^ cells per well. After 3 h of co-culture, the cells were harvested for subsequent CCK-8 viability assays and analysis of the IgA^+^ B cell fraction. The supernatant was collected for IgA measurement via ELISA.

**Cell viability**

The CCK8-Detection kit (CT01a, Cellcook) was used to determine primary B cell viability following the manufacturer’s protocol. The colorimetric reaction was measured at OD = 450 nm using a SmartReader™ 96 Microplate Absorbance Reader (Bio-Rad, Hercules, CA).

**Flow cytometry assay**

The following antibodies were used: Mouse Anti-Chicken Bu-1-AF647 (8395-30, Southern Biotech), FITC-anti-mouse IgA mAb (4204, Invitrogen), CD19-PE (115507 and 302254, Biolegend), CD138-BV421 (356515, Biolegend), and IgA-PE (333503, Biolegend). The concentration of cell suspension was determined using an automated cell counter (TC20, Bio-Rad). Cells were then surface-stained with antibody at 4 °C for 20 min. Staining cell proportions were quantified using a FACSCalibur flow cytometer (Becton Dickinson, Palo Alto, CA) and Cell Quest software (Becton Dickinson, Franklin Lakes, China).

**Quantification of** **cecal and serum IgA concentrations**

IgA concentrations in the supernatant of co-culture, cecal content, and serum were measured using chicken and mouse IgA ELISA Kit (Bethyl) following the manufacturer’s instructions. The absorbance of each well was read at 450 nm with a microplate reader (Bio-Rad).

**qPCR analysis of the gene expression**

The RNAiso Plus reagent (Takara Bio, Japan) was used to extract total RNA of Ramos cells, and reverse transcript into cDNA by PrimeScriptTM IV 1st strand cDNA Synthesis Mix (Takara Bio). The primers used as follow: *Gapdh* F (5′‐TGTAGTTGAGGTCAATGAAGGG‐3′) and R (5′‐ACATCGCTCAGACACCATG‐3′), *Blimp1* F (5′‐CAGAGTTCATTTTTCTCAGTGCTC‐3′) and R (5′‐GAAAGGCTTCACTACCCTTATCC‐3′), *Xbp1s* F (5′-GAGTCCGCAGCAGGTG-3′) and R (5′-TCCTTCTGGGTAGACCTCTGGGAG-3′), *Igha1* F (5′‐GACCCCGCTAACCGCCACC‐3′) and R (5′‐CGTCAGCGTCACCAGCTCGTT‐3′). The gene expression was determined using the 2^-△△CT^ method.

**Statistical analysis**

Statistical analyses were performed using GraphPad Prism or R software (v3.4.1). Statistical significance in microbial abundance and diversity between two groups was assessed using the Wilcoxon rank-sum test. Unless otherwise specified, comparisons between two groups were performed using an unpaired, two-tailed Student’s *t*-test, while one-way Analysis of Variance (ANOVA) was applied for comparisons among three groups. Results are presented as mean ± SD, with statistical significance indicated by * *p* < 0.05, ** *p* < 0.01, and *** *p* < 0.001. Sample sizes and statistical tests are mentioned in the figure legends.

**REFERENCES**

1. Patience, John F., Mariana C. Rossoni-Serão, and Néstor A. Gutiérrez. 2015. “A review of feed efficiency in swine: biology and application.” *Journal of Animal Science and Biotechnology* 6: 33. <https://doi.org/10.1186/s40104-015-0031-2>

2. Zou, Xian, Tianfei Liu, Ying Li, Peng Chen, Xia Yan, Jie Ma, Jian Ji, et al. 2023. “Long-term divergent selection for residual feed intake in Chinese broiler chickens.” *Poultry Science* 102: 102298. <https://doi.org/10.1016/j.psj.2022.102298>

3. Liu, Binghang, Jianying Yuan, Siu-Ming Yiu, Zhenyu Li, Yinlong Xie, Yanxiang Chen, Yujian Shi, et al. 2012. “COPE: an accurate k-mer-based pair-end reads connection tool to facilitate genome assembly.” *Bioinformatics* 28: 2870-2874. <https://doi.org/10.1093/bioinformatics/bts563>

4. Edgar, Robert C. 2013. “UPARSE: highly accurate OTU sequences from microbial amplicon reads.” *Nature Methods* 10: 996-998. <https://doi.org/10.1038/nmeth.2604>

5. Edgar, Robert C., Brian J. Haas, Jose C. Clemente, Christopher Quince, and Rob Knight. 2011. “UCHIME improves sensitivity and speed of chimera detection.” *Bioinformatics* 27: 2194-2200. <https://doi.org/10.1093/bioinformatics/btr381>

6. Cole, James R., Qiong Wang, Jordan A. Fish, Benli Chai, Donna M. McGarrell, Yanni Sun, C. Titus Brown, Andrea Porras-Alfaro, Cheryl R. Kuske, and James M. Tiedje. 2014. “Ribosomal Database Project: data and tools for high throughput rRNA analysis.” *Nucleic Acids Research* 42: D633-642. <https://doi.org/10.1093/nar/gkt1244>

7. Caporaso, J. Gregory, Justin Kuczynski, Jesse Stombaugh, Kyle Bittinger, Frederic D. Bushman, Elizabeth K. Costello, Noah Fierer, et al. 2010. “QIIME allows analysis of high-throughput community sequencing data.” *Nature Methods* 7: 335-336. <https://doi.org/10.1038/nmeth.f.303>

8. Douglas, Gavin M., Vincent J. Maffei, Jesse R. Zaneveld, Svetlana N. Yurgel, James R. Brown, Christopher M. Taylor, Curtis Huttenhower, and Morgan G. I. Langille. 2020. “PICRUSt2 for prediction of metagenome functions.” *Nature Biotechnology* 38: 685-688. <https://doi.org/10.1038/s41587-020-0548-6>


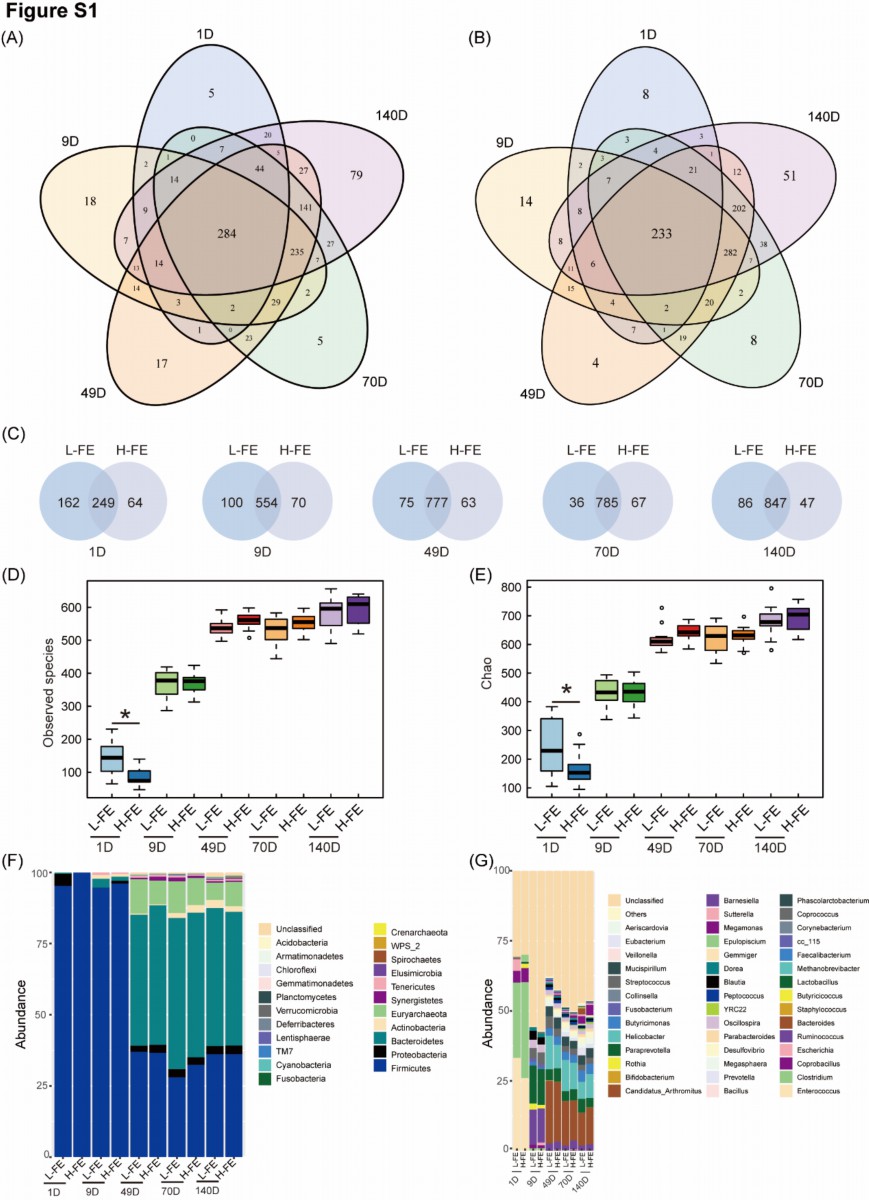


**Figure S1 Dynamic alterations in the microbial community during the growth of low feed efficiency (L-FE) and high feed efficiency (H-FE) chickens.** (A and B) Venn diagrams depict the operational taxonomic units (OTUs) at different ages of L-FE and H-FE chickens (*n* ≥ 9). (C) Venn diagrams for OTUs between L-FE and H-FE chickens at different time points. (D and E) Measurement of alpha diversity using observed species and chao index, and statistical significance was using Wilcoxon rank-sum test and represented by * *p* < 0.05. (F and G) Microbial composition of L-FE and H-FE chickens at phylum and genus levels.


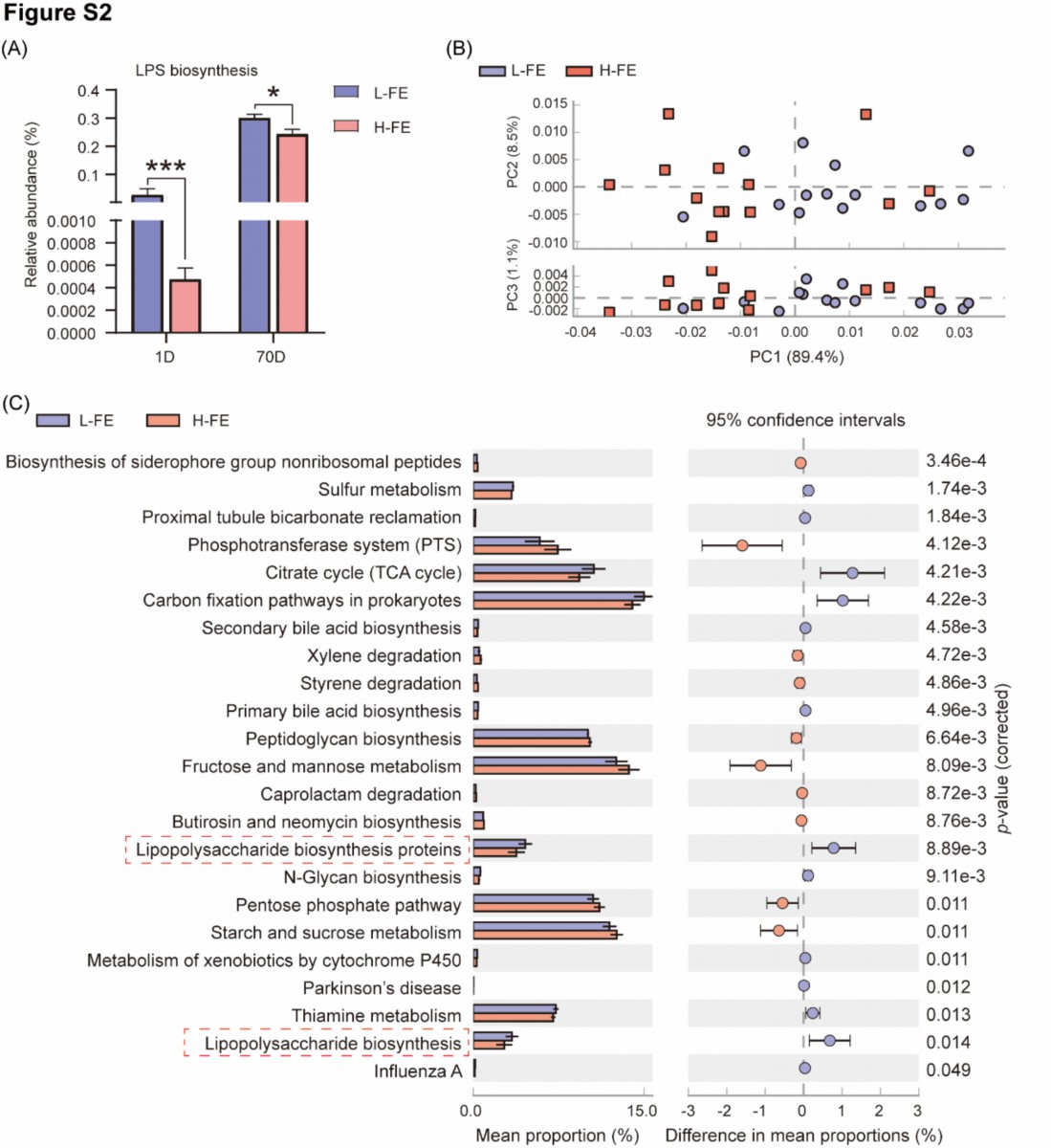


**Figure S2 Differential functions analysis of the cecal microbiota based on** **Phylogenetic Investigation of Communities by Reconstruction of Unobserved Species (PICRUSt) prediction between L-FE and H-FE chickens.** (A) Relative abundance of bacteria related to lipopolysaccharide (LPS) biosynthesis based on PICRUSt prediction. (B) Scatterplot generated by principal component analysis (PCA) using PICRUSt, revealing the diversity of bacterial functions in 70-day-old chickens. (C) Predictive function analysis of the microbiome by PICRUSt at day 70. The statistical significance was analyzed using statistical analysis of metagenomic profiles (STAMP) software, applying a two-sided White’s non–parametric *t*-test on. Asterisks denote significance levels: * *p* < 0.05, *** *p* < 0.001.


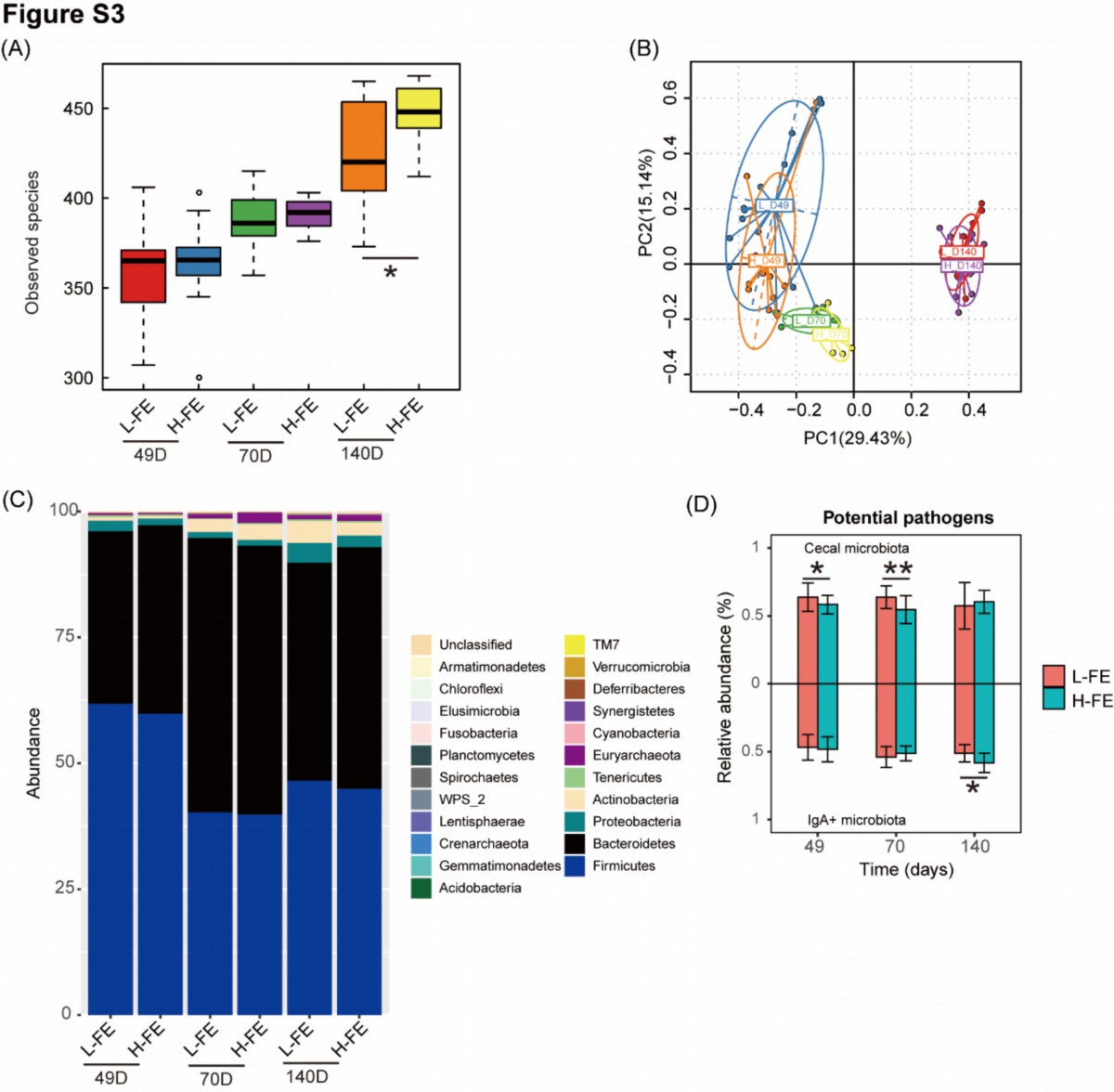


**Figure S3 Composition and function of immunoglobulin A (IgA)-coated microbiota between L-FE and H-FE chickens.** (A) Comparison of the observed OTUs between L-FE and H-FE chickens (*n* ≥ 7). (B) PCA plot based on weighted UniFrac distances. (C) Composition of IgA-coated bacterial communities at the phylum level. (D) Prediction of potential pathogens in the cecal microbiome and IgA^+^ microbiome using BugBase. Statistical significance of alpha diversity and relative abundance was analyzed using a Wilcoxon rank-sum test. Asterisks denote significance levels: * *p* < 0.05, ** *p* < 0.01.


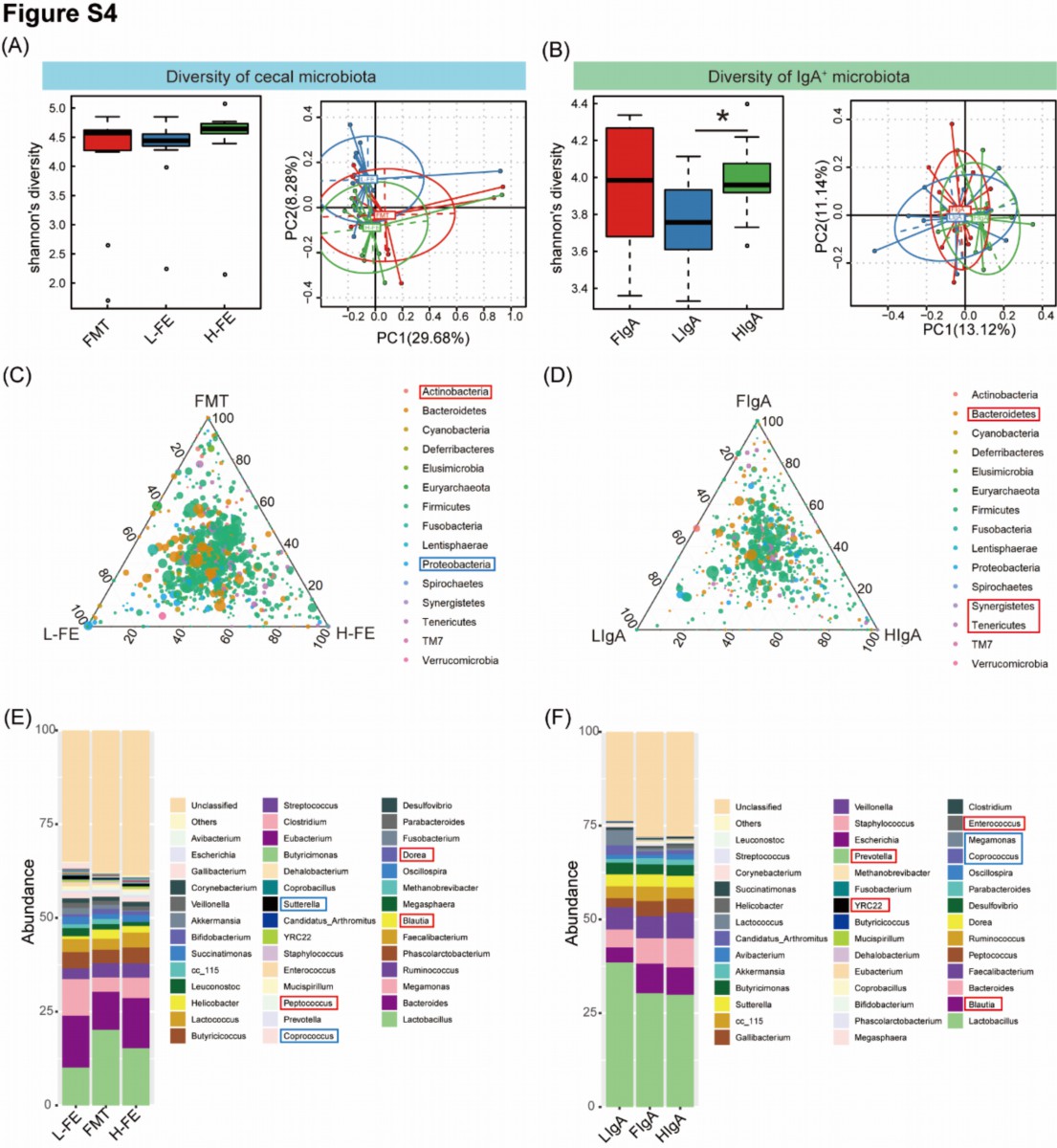


**Figure S4 Effects of fecal microbiota transplantation (FMT) on cecal and IgA-coated microbial diversity and composition.** (A and B) Microbial diversity depicted through Shannon’s diversity index and PCA plot based on weighted UniFrac distances for the cecal microbiota (*n* ≥ 14) (A) and IgA^+^ microbiota (*n* ≥ 11) (B). (C and D) Bacterial community composition at the OTU level of the cecal and IgA-coated microbiomes. (E and F) Bacterial community composition at the genus level for cecal and IgA-coated microbiota. Relative abundance in FMT and H-FE was significantly higher (red box) or lower (blue box) compared to that in L-FE, as determined by a Wilcoxon rank-sum test with Benjamini-Hochberg FDR correction.


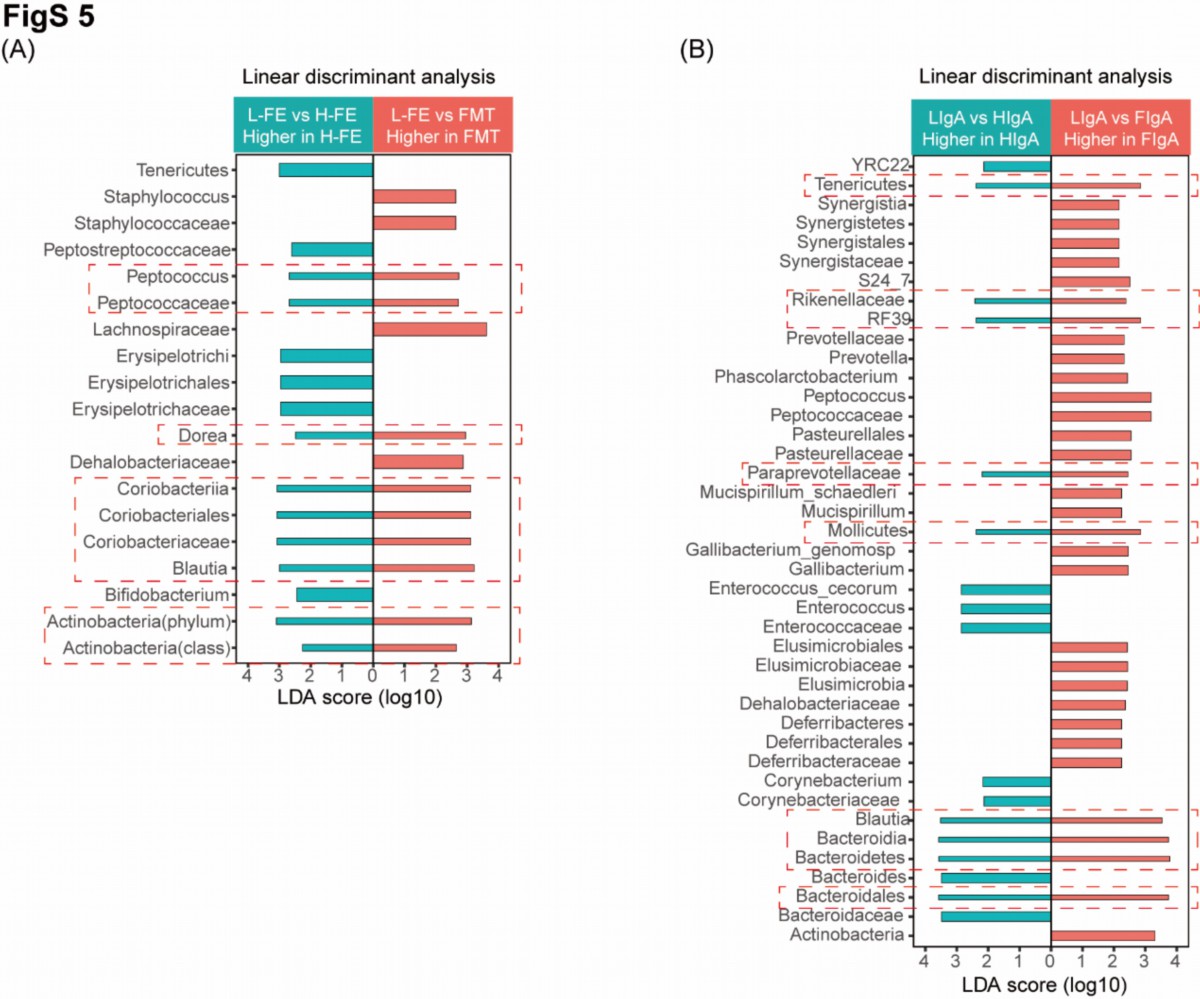


**Figure S5 Identification biomarker bacteria associated with feed efficiency.** (A and B) Linear discriminant analysis (LDA) for the most discriminant bacterial taxa identified by linear discriminant analysis effect size (LEfSe) in the cecal microbiota (A) and IgA^+^ microbiota (B).


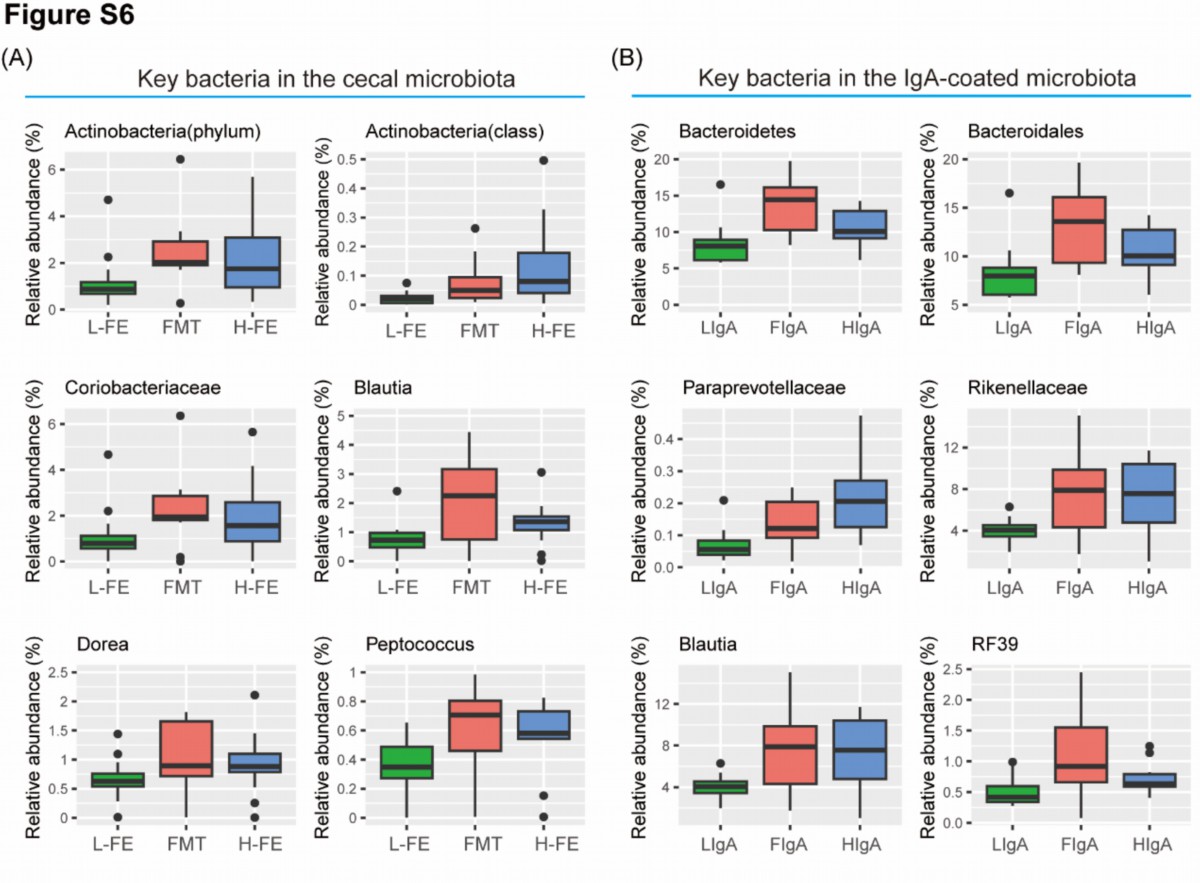


**Figure S6** **Boxplots display the relative abundance of shared bacterial taxa identified by LDA score analysis.** (A) Key bacterial strain in the cecal microbiome. (B) Key bacterial strain in the IgA^+^ microbiome. The bar length represents the log10-transformed linear discriminant score.


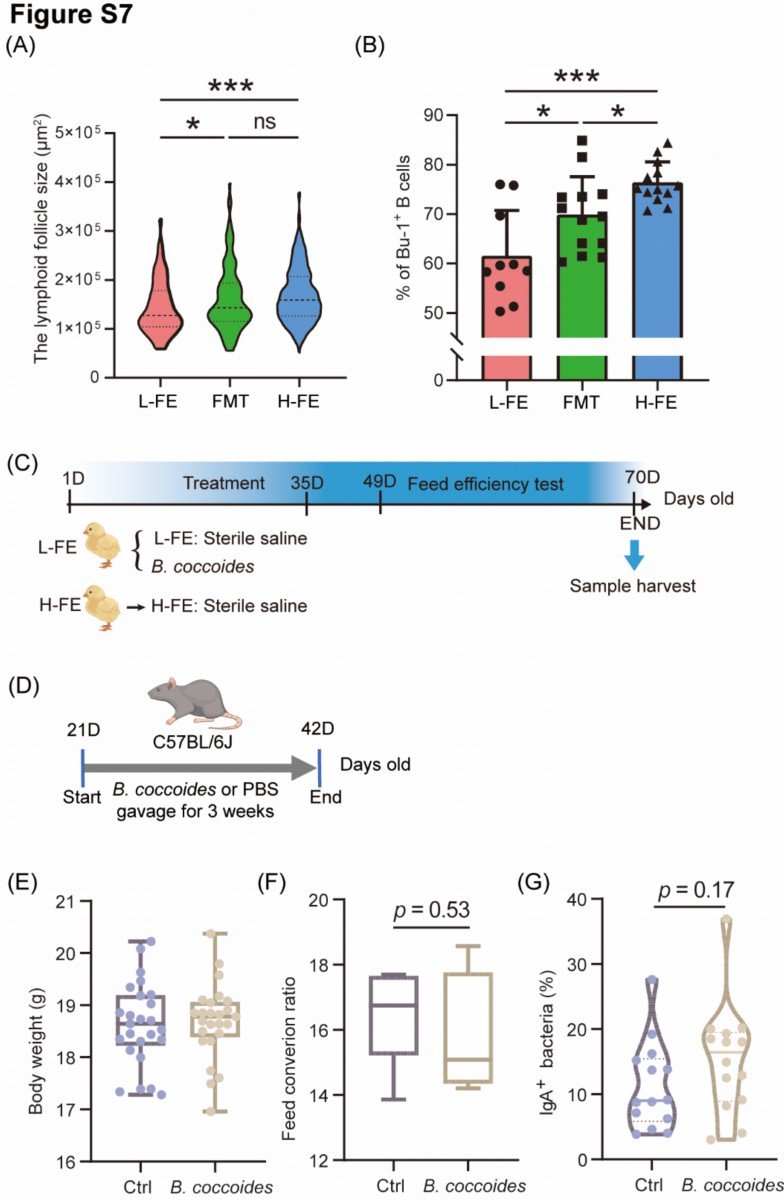


**Figure S7 FMT on chickens and *Blautia coccoides* administration in chickens and mice.** (A) Bursal lymphoid follicle size determined through hematoxylin and eosin (H&E) staining and measured using light microscopy. (B) Quantification percentage of bursal Bu-1^+^ B cell via flow cytometry. (C) Experimental procedures in chickens (*n* ≥ 25). (D) Experimental procedures in mice (*n* = 25). (E) Body weight of mice. (F) Feed conversion ratio (*n* ≥ 4). (G) The proportion of IgA^+^ bacteria in cecal contents (*n* = 15).


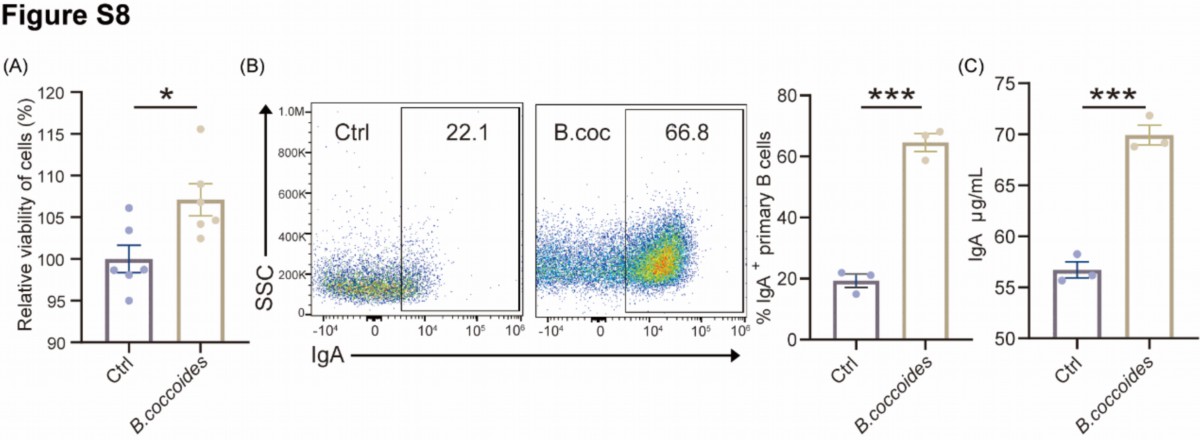


**Figure S8 Primary B cells of chicken co-culture with *B. coccoides*.** (A) Cell viability determined by CCK8 assay. (B) Percentage of IgA^+^ B cells post-co-culture. (C) Concentration of IgA. Statistical significance was analyzed using an unpaired two-tailed Student’s *t* test. Asterisks denote significance levels: * *p* < 0.05, *** *p* < 0.001. SSC, side scatter.
